# Phytochemical Profile, Toxicity Evaluation and Antinociceptive Effect of the *n*-Butanolic Fraction from the Leaves of *Calotropis procera* (Aiton) W.T Aiton (Apocynaceae)

**DOI:** 10.3390/plants14233622

**Published:** 2025-11-27

**Authors:** Kailane Lourenço Araújo, Natanael Teles Ramos de Lima, Pedro Artur Ferreira Marinho, Dara Rayanne da Silva Guedes, Marcelo Sobral da Silva, Yuri Mangueira do Nascimento, Josean Fechine Tavares, José Maria Barbosa Filho, Cinthya Maria Pereira de Souza, Vanda Lúcia dos Santos, Harley da Silva Alves, Ivana Maria Fechine, Alisson Macário de Oliveira

**Affiliations:** 1 Departamento de Farmácia, Centro de Ciências Biológicas e da Saúde, Universidade Estadual da Paraíba, Campina Grande 58429-500, PB, Brazil; 2 Departamento de Farmácia, Centro de Ciências da Saúde, Universidade Federal da Paraíba, Joao Pessoa 58051-900, PB, Brazil

**Keywords:** “Silk cotton”, *n*-butanolic fraction, HPLC-ESI-MS/MS, roseoside, acute toxicity, antinociceptive activity

## Abstract

*Calotropis procera*, known as “Silk cotton”, stands out for the presence of various classes of bioactive compounds responsible for its ethnopharmacological properties. The study aimed to conduct a phytochemical investigation, evaluating the in vitro and in vivo toxicity together with the antinociceptive potential of an n-butanolic fraction (FB) from the leaves. The crude ethanolic extract (CEE) was obtained by maceration in ethanol for 72 h. It was then partitioned using a gradual solvent sequence. The FB was analyzed by HPLC-ESI-MS/MS in negative mode and ^1^H and ^13^C NMR. Toxicity was assessed by the erythrocyte hemolytic assay and acute oral toxicity test at a single dose of 300 mg·kg^−1^. The antinociceptive effect was assessed by the acetic acid-induced abdominal writhing test and the formalin test in mice at doses of 3.75, 7.5 and 15 mg·kg^−1^ *per os*. HPLC-ESI-MS/MS analysis identified flavonoids, phenolic acids, and the megastigmane roseoside, isolated for the first time in *C. procera*. The FB did not cause hemolytic effects or behavioral or physiological changes in mice. It showed an antinociceptive effect at all doses, reducing abdominal writhing by up to 91.46% and the licking time in phases 1 and 2 of the formalin test by up to 63.83% and 91.73%, respectively. In this study, it was possible to determine that an FB of a crude extract of *C. procera* leaves has antinociceptive activity, possibly associated with the phenolic compounds and roseoside found, with a lack of toxicity *in vitro* and *in vivo*, validating its ethnopharmacological use.

## 1. Introduction

The use of plant based medicines dates back thousands of years [1]. In the period from 1981 to 2019, natural products stood out as a promising source of bioactive compounds for the production of drugs, with the aim of treating numerous diseases. They also remain among the main agents involved in the synthesis of new molecules with pharmacological potential [2]. The use of processes that enable the faster isolation and identification of secondary metabolites is of utmost importance, as they facilitate toxicological studies for determining safe usage doses, as well as ethnopharmacological studies, thus accelerating drug development [3].

Thus, Apocynaceae is one of the largest families in the plant kingdom, with notable representation among angiosperms, widespread across all continents except Antarctica, and possessing high biological potential [4]. Belonging to this family, the shrub *Calotropis procera* (Aiton) W.T Aiton stands out, popularly known in Brazil as “Algodão de seda” (“Silk cotton”). *C. procera* is used in various traditional medicine systems, such as Ayurveda, Unani, Arabic, and Indo-Sudanese medicine, with its ethnomedicinal use widely associated with the treatment of edema, taeniasis, laxative effects, relief of abdominal pain, and asthma [5]. In addition, its leaves are used in folk medicine to treat joint pain, minimize swelling, and stem bark powder for malaria, elephantiasis, and leucoderma [6].

Furthermore, *C. procera* is pharmacologically relevant, as studies support its biological activities, such as hepatoprotective, antinociceptive, anti-inflammatory, antitumor, antimicrobial, antioxidant, anticonvulsant, and neuroprotective effects [7,8,9]. The phytocompounds flavonoids, alkaloids, saponins, terpenes, cardenolides, and steroids present in the various parts of the plant (roots, leaves, flowers, latex, stem) are considered to be responsible for these properties [10,11,12]. However, studies on its antinociception properties are scarce in the literature, especially those related to fractions obtained from it.

Compounds of plant origin have been recognized for their considerable analgesic properties, with few adverse effects [13,14]. For example, many flavonoids are becoming candidates for new natural painkillers [15]. In addition, they are considered therapeutic substitutes to modulate nociception, since opioid, non-opioid, and anti-inflammatory analgesic drugs available in current therapy are often associated with adverse effects such as addiction, psychomotor impairment, and gastrointestinal and cardiovascular damage [16,17,18]. In this sense, studying new pharmacological options from medicinal plants is a promising alternative [19].

Thus, this study carried out a phytochemical investigation, evaluation of the in vitro and in vivo toxicological profile (behavioral, hematological, and biochemical parameters) and the antinociceptive potential of the *n*-butanolic fraction obtained from the crude ethanolic extract of *C. procera* leaves, considering its popular use in the treatment of pain and inflammation, as well as the scarcity of chemical and biological data in the literature about this fraction.

## 2. Results

### 2.1. Phytochemical Characterization by HPLC-ESI-MS/MS

The FB was analyzed by high-performance liquid chromatography coupled with mass spectrometry (HPLC-MS), with the aim of annotating the compounds present in it and, consequently, contributing to the chemotaxonomy of the genus. The elution gradient used in the chromatographic analyses was able to separate compounds belonging to secondary metabolite classes such as phenolic acids, flavonoids, their glycosylated derivatives, and a substance from the megastigman class. The annotation of all the compounds was based on the fragmentation profile in comparison with data from the literature.

**Figure 1:**
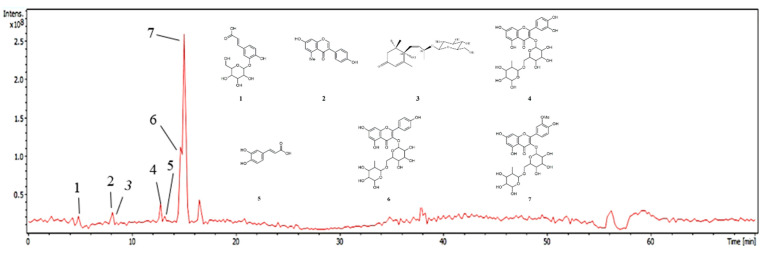
Chromatogram of the FB base peak from *C. procera* leaves. Numbers (1–7) refer to the peaks in Table 1.

As a result, 7 peaks were noted on the base peak chromatogram of the fraction (Figure 1) and the tentatively identified compounds can be seen in Table 1, as well as their proposed chemical structure in Figure 1. Among the phenolic acids, flavonoids and their glycosylated derivatives, the following stand out: caffeic acid (179 *m*/*z*), its glycoside caffeic acid-*O*-hexoside (377 *m*/*z* (341-H+Cl)), quercetin-*O*-rutinoside (609 *m*/*z*), kaempferol-*O*-rutinoside (593 *m*/*z*), isorhamnetin-*O*-rutinoside (623 *m*/*z*), and methyldaidzein (276 *m*/*z*), respectively, and the megastigmane roseoside (431 *m*/*z* (385-H+Formate)), not yet isolated in the species.

### 2.2. Isolation and Characterization of Compounds

Using preparative HPLC, it was possible to isolate Compound **4** from fraction FB3, which appeared as an amorphous brown solid, weighing 9 mg. The ^1^H NMR spectrum (DMSO-d_6_, 400 MHz) showed the presence of diastereotopic hydrogens [δ_H_ 2.07 (1H, *d*, *J* = 16.8) and 2.41 (1H, *d*, *J* = 16.8)], signals at δ_H_ 5.74 (1H, *s*, H-4), δ_H_ 5.77 (1H, *d*, *J* = 5.2, H-7), and δ_H_ 5.78 (1H, *t*, H-8) corresponding to olefinic hydrogens (Table S1), the latter two (H-7 and H-8) indicating a *cis* exocyclic olefinic bond; and singlets integrating for 3H, suggestive of three methyl groups (Me), with chemical shifts at δ_H_ 0.92 (H-12), δ_H_ 0.93 (H-11), and δ_H_ 1.81 (H-13). In addition, a doublet suggestive of an anomeric hydrogen was observed at δ_H_ 4.16 (1H, *d*, *J* = 7.8), integrating for 1H, which indicates the presence of a sugar moiety in the structure (Figures S3–S6).

The ^13^C NMR spectrum (DMSO-d_6_, 100 MHz), obtained using the Broad-Band (BB) technique, supports the previously obtained data, suggesting the presence of methyl groups [δ_C_ 24.1 (C-12), 23.1 (C-11), 18.9 (C-13)], which, along with the chemical shift in H-13, indicate that Me-13 is attached to an olefinic carbon. Additionally, a doublet integrating for 3H was observed, assigned to δH 1.10 (H-10), along with a signal at δ_C_ 20.9 (C-10), corresponding to Me-10. A signal at δ_C_ 197.5 (C-3) was also observed, suggestive of an α,β-unsaturated carbonyl group (Figure S7).

Analysis using two-dimensional direct heteronuclear correlation spectra (HSQC) made it possible to assign chemical shifts related to hydrogenated carbons. This revealed correlations between C-11 (δ_C_ 23.1) and H-11 (δ_H_ 0.93), C-12 (δ_C_ 24.1) with H-12 (δ_H_ 0.92), C-13 (δ_C_ 18.9) correlating with H-13 (δ_H_ 1.81) and C-9 (δ_C_ 73.7) with H-9 (δ_H_ 4.30) (Figure S8).

**Figure 2:**
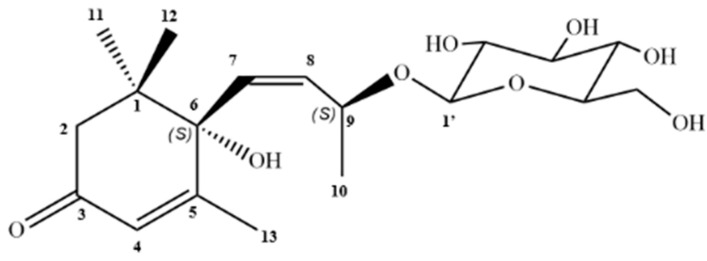
Structure of roseoside isolated from *C. procera*.

The data from the contour map of the two-dimensional heteronuclear correlation spectrum (HMBC) shows the correlation of the diastereotopic hydrogens (H-2a and H-2b) with C-3, thus determining the position of the carbonyl on carbon 3. Me-11 and Me-12 showed correlations with carbons C-1 (δ_C_ 40.96), C-2 (δ_C_ 49.39) and C-6 (δ_C_ 77.9), proving their connection to C-1, while Me-10 and Me-13 correlated with C-8, C-9 and C-3, C-4, C5 and C-6, respectively (Figure S9; Table S2). In light of these considerations, the data indicate that CP-R corresponds to megastigmane roseoside (Figure 2).

### 2.3. Hemolytic Activity

The hemolytic potential of FB was evaluated using the experimental hemolysis model commonly employed for the analysis of plant extracts. FB did not induce significant rupture of erythrocyte membranes at the concentrations used in the test. The highest concentration dose (2000 µg·mL^−1^) showed a 1.3% hemolytic rate, while the lowest concentration (125 µg·mL^−1^) did not induce hemolysis, with a rate of 0%. The intermediate doses (250, 500 and 1000 µg·mL^−1^) also showed no erythrocyte destruction

### 2.4. Acute Oral Toxicity Test

The single dose of 300 mg·kg^−1^ administered orally of the FB of *C. procera* leaves did not cause any mortality. The pharmacological screening showed an increase in ambulation in the first 15 min, after which there were no behavioral changes. With regard to the physiological parameters of feed and water consumption and absolute evolution, treatment with FB did not cause any changes when compared to the control (Table 2).

Similarly, to the physiological parameters, the hematological analyses of the animals submitted to the acute oral toxicity test showed no changes in the erythrocyte count or morphology, and the single dose of FB (300 mg·kg^−1^) did not cause any changes in the white series (WBC, SEG, LIN, MON, BASO and EOS) when compared to the control group, as shown in Table 3.

Regarding the biochemical parameters of the serum collected from the mice, there were also no variations in the markers of liver and kidney function or other general markers in the group treated with FB (300 mg·kg^−1^), compared to the control group, as shown in Table 4.

### 2.5. Antinociceptive Evaluation

#### 2.5.1. Acetic Acid Induced Writhing Test

**Figure 3:**
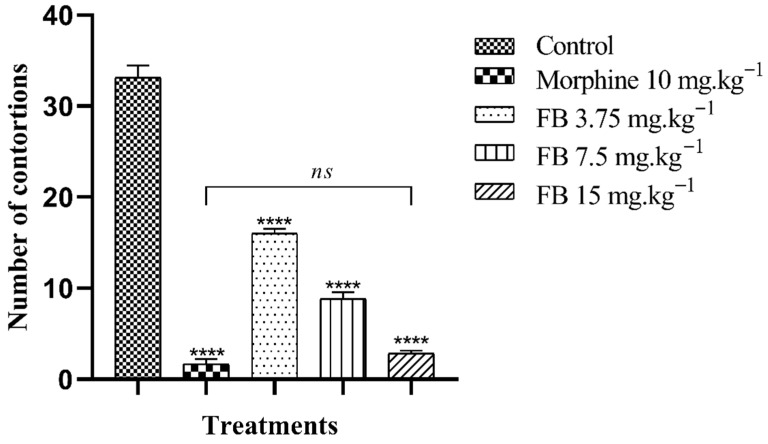
Antinociceptive effect of the *n*-butanolic fraction in the abdominal writhing test. The bars represent the mean number of abdominal contortions ± SEM. (****) indicates a significant difference (*p* < 0.001) when compared to the control. (*ns*) indicates no significant difference (*p* > 0.05) in relation to morphine, one-way analysis of variance (ANOVA) followed by Tukey’s test.

Oral administration of FB at all the doses tested significantly (*p* < 0.001) reduced the animals’ nociceptive behavior in a dose-dependent manner. Thus, there was a reduction in the number of abdominal contortions induced by acetic acid with percentages of 51.76, 73.38 and 91.46%, referring to the doses of 3.75, 7.5 and 15 mg·kg^−1^ of FB, respectively, while the group treated with morphine 10 mg·kg^−1^ showed a 94.97% inhibition when compared to the control group. It should be noted that treatment with the highest dose of FB (15 mg·kg^−1^) did not result in a statistical difference in the number of contortions compared to morphine, the standard drug used, which reinforces the species’ potential for this activity (Figure 3).

#### 2.5.2. Formalin Test

The oral treatment of FB from *C. procera* leaves showed the potential to inhibit nociception in the neurogenic and inflammatory phases, but with a more significant result in the second phase. In the first phase (neurogenic), treatment with 3.75, 7.5 and 15 mg·kg^−1^ of FB reduced hind paw licking time by 14.59, 42.23 and 63.83%, respectively, when compared to the control group. The reference drugs, morphine (10 mg·kg^−1^) and indomethacin (20 mg·kg^−1^), suppressed licking time by 90.57 and 31.00%, in that order.

**Figure 4:**
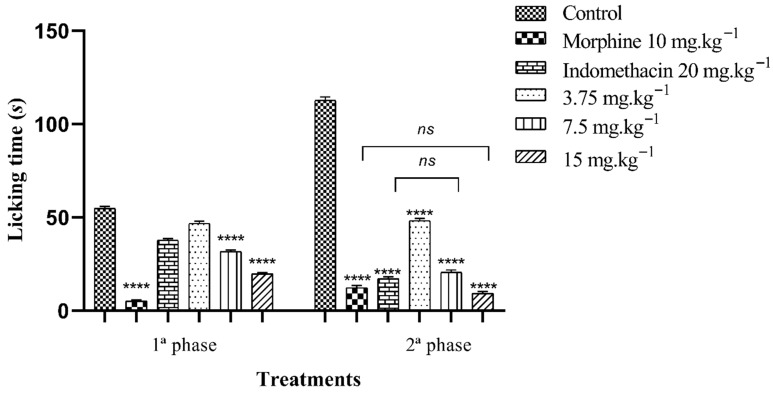
Antinociceptive effect of the n-butanolic fraction in the formalin test. The bars represent the average time spent by the mice licking their paws ± SEM. (****) indicates a significant difference (*p* < 0.001) when compared to the control. (*ns*) indicates no significant difference (*p* > 0.05) when compared to morphine or indomethacin, one-way analysis of variance (ANOVA) followed by Tukey’s test.

In the second phase of the test (inflammatory), treatment with 3.75, 7.5 and 15 mg·kg^−1^ reduced the time the animals spent licking their paws by 51.17, 81.68 and 91.73%, respectively, compared to the control group. The standard drugs, morphine (10 mg·kg^−1^) and indomethacin (20 mg·kg^−1^), inhibited nociception by 89.07 and 84.79% (Figure 4).

## 3. Discussion

The use and integration of processes aimed at isolating and identifying secondary metabolites more quickly is of the utmost importance, as it optimizes ethnopharmacological research and, consequently, the discovery of new drugs. In this sense, this study provided phytochemical information on the *n*-butanolic fraction of the *C. procera* species, based on its chemical characterization and isolation of compounds, as well as pharmacological information, showing the absence of toxicity in vitro and in vivo in a single dose and strong activity in experimental models of nociception, making it a therapeutic option.

*C. procera* plays a significant role in ethnopharmacology due to its diversity of bioactive compounds and consequent biological activity. Extracts, fractions, and substances isolated from the species have been researched for their antiepileptic, antiulcerogenic, antiasthmatic, anti-inflammatory, anticancer, antimicrobial, and antipyretic properties, among others [29]. In addition, it is widely used in the commercial and industrial areas for biofuel production, since it is rich in hydrocarbons, biopesticides, due to its latex, as well as being used in cheese making [30].

In view of this, the HPLC-ESI-MS/MS of the FB showed phenolic acids, flavonoids, their glycosylated derivatives and a compound from the megastigmane class, which have antioxidant, anti-inflammatory and anti-tumor activities, as well as being associated with the antinociceptive activity of the FB [9,31,32,33,34,35]. Compound **1** showed a diagnostic ion at 179 *m*/*z* and at 161 *m*/*z*, compound **5** at 179 *m*/*z*, which suggests the existence of a cinnamic acid derivative, such as caffeic acid. In addition, the loss of 162 mass units indicates the presence of a hexose in molecule **1** [20,26]. In addition, for compound **1**, this was noted due to the presence of trace mineral residues adsorbed onto the glassware surfaces, even after rigorous cleaning procedures. Furthermore, the mass spectrum exhibits an isotopic pattern characteristic of the presence of a chlorine atom, as evidenced by the ion at *m*/*z* ~377/379. Compound **2** was annotated by its fragment ion at 267 *m*/*z* in comparison with a study in the literature [21], which identified 7,2′-dihydroxy-4′-methoxyl isoflavan, better known as methyldaidzein, as well as the previous substances, **3,** with a retention time of 8.2 min, which was attributed to megastigman roseoside in analogy to fragmentation patterns present in the literature [23].

Compound **4** showed a deprotonated aglycone fragment at 301 *m*/*z*, which suggests its origin from quercetin, and the loss of 308 Da indicates the existence of the rutinoside osidic unit in its structure [25]. Substance **6,** with a retention time of 14.7 min, showed a deprotonated molecular ion at 593 *m*/*z*, an absence of 308 Da (rutinoside) and an ion with maximum intensity at 285 *m*/*z*, suggestive of the aglycone [27]. For compound **7**, a deprotonated aglycone fragment was observed at 315 *m*/*z*, which is suggestive of the isorhamnetin molecule, as well as the ions at 300, 271 and 255 *m*/*z*, which corroborate the data and the loss of 308 Da indicates the presence of the rutinoside sugar in the structure [28].

Similarly, from the methanolic extract of the leaves of *C. procera* it was possible to isolate quercetin, quercetin-*O*-rutinoside, isoquercetin, isorhamnetin-*O*-rutinoside, and kaempferol-*O*-rutinoside [9]. From the methanolic leaf extract, the presence of rutin, isorhamnetin-robinoside, isorhamnetin-rutinoside, kaempferol-rutinoside, kaempferol-hexoside, quercetin, and afroside was also reported, showing similarity to the chemical profile identified in the FB of the leaves in the present study. Additionally, in the aqueous extract of the leaves, compounds such as catechin, rutin, *p*-coumaric acid, and kaempferol were identified, with *p*-coumaric acid being the most abundant [35]

Furthermore, the butanolic fractions typically contain compounds with higher polarity, such as phenolic acids, flavonoids, tannins, and their glycosides, which supports the data mentioned above [36]. The investigation of butanolic fractions involves analytical challenges due to their high polarity; however, this fraction constitutes a plant matrix of great interest, as it concentrates bioactive compounds of pharmacological relevance, as observed. Furthermore, the limited availability of studies focused on this fraction in the species under analysis reinforces the relevance of its choice in this research, which also complements the results obtained with fractions obtained by other solvents. In this context, these findings highlight the abundance of molecules from different classes present in the FB of the species under study, with previously described pharmacological effects, underscoring its potential as a source for new chemical and biological discoveries.

In addition, based on the information obtained by HPLC-ESI-MS/MS of the FB, the fragmentation pattern of roseoside was observed, which had not previously been identified in the species *C. procera*. Compound **4**, labeled as CP-R, from the FB3 fraction, was isolated as a brown amorphous solid, weighing 9 mg, and possessing two stereogenic centers at C-6 and C-9 [37]. Thus, in comparison with the scientific data, which exhibit chemical shift signals that closely correspond to those reported in the literature, including the aforementioned stereogenic center, the α,β-unsaturated carbonyl, the unsaturation between C-2 and C-3, as well as the characteristic C_13_ carbon skeleton and the *O*-glycosidic linkage to glucose. In this context, CP-R was identified as the roseoside, corroborating the data from the phytochemical characterization of FB [38,39,40,41]. CP-R was identified as the roseoside, corroborating the data from the phytochemical characterization of FB [36,37,38,40].

The roseoside (C_19_H_30_O_8_) has previously been isolated from the leaves of species such as *Corchorus olitorius* L. (Malvaceae), *Piper crocatum* (Piperaceae), *Antidesma bunius* (L.) Spreng (Phyllanthaceae), *Ficus callosa* (Moraceae), and *Euodia meliaefolia* (Rutaceae), as well as from the stem of *Kandelia candel* (Rhizophoraceae) and the aerial parts of *Gynura bicolor* (Willd.) DC (Asteraceae) and *Sauropus androgynus* (Phyllanthaceae) [41]. However, this compound is reported here for the first time from *C. procera*.

With the determination of the phytochemical profile and considering the ethnopharmacological data available for the species, the safety assessment of the FB was carried out following the recommendations of the World Health Organization, which advocates for the toxicological evaluation of medicinal plants and herbal medicines to establish a safe dosage prior to consumption [42]. Thus, one of the toxicity screening tests commonly used for initial evaluation is hemolysis. This is capable of indicating toxic events that could cause damage to the erythrocyte membrane, in which hemolysis rates of less than 10% indicate that the substance is safe for experimental models [43,44].

As a result, FB did not induce significant hemolysis, in which at its highest concentration tested (2000 µg·mL^−1^) it showed a hemolytic index of 1.3%, which corroborates its safe use for in vivo preclinical trials. In addition, flavonoids and phenolic acids, which make up the majority of FB, are less associated with hemolysis than other groups of metabolites, such as saponins and triterpenes [45,46].

Subsequent to confirming safety in the hemolysis assay, the study proceeded to the acute oral toxicity test in mice to evaluate in vivo safety. Following a single oral dose of 300 mg·kg^−1^, the FB from *C. procera* leaves was found to be safe for use, as no mortality or significant changes (*p* > 0.05) were observed in food and water intake or absolute weight gain. Reductions in these parameters and in animal development are indicative of systemic toxicity [47].

In addition to the physiological parameters, the hematological indices were evaluated as they are fundamental indicators for monitoring and preventing adverse effects of drugs and other chemical substances [48]. The analysis revealed that single-dose oral treatment with 300 mg·kg^−1^ of FB did not cause any changes in the red series (hematocrit, hemoglobin, MCV, MCH, MCHC) or in the leukocyte count (segmented, lymphocytes, monocytes, eosinophils). Similarly, hepatic enzymatic and renal biomarkers are used to assess the integrity of the liver and kidneys, respectively [49]. The enzymatic biochemical parameters (AST, ALT, GGT, ALP, albumin) and renal function (creatinine and urea) remained unchanged compared to the control group. In this sense, considering the absence of significant changes, FB is a substance with an LD_50_ > 300 mg·kg^−1^.

Similarly, the acute and subacute toxicity of the ethanolic extract of *C. procera* flowers, obtained by the maceration method, was evaluated with the aim of proving the use of the species as a safe and effective treatment. In this context, it was observed that during the 14-day study, there was no mortality, no behavioral abnormalities, and no hematological and biochemical alterations in relation to the single dose of 2000 mg·kg^−1^ in the mice tested (*Swiss* albinos, *Mus musculus*). In the subacute test, the doses of 300, 1000, and 2000 mg·kg^−1^ of the ethanolic extract of the flowers also showed no deaths, and the hematological and biochemical parameters remained within the expected normal range when compared to the control [50].

The use of plants, one of the main examples of natural products, as analgesic agents in traditional medicine is an ancient practice, with an estimated 70,000 plant species used ethnomedicinally worldwide [51]. To evaluate the antinociceptive activity of *C. procera* FB, two preclinical experimental methods commonly used to analyze the antinociceptive properties of natural matrices were used: abdominal contortions induced by acetic acid and the formalin test.

In the abdominal writhing test, it is possible to assess the nociceptive action of substances in a non-specific way, since antihistamine, narcotic, and anxiolytic compounds can be active in this test [52,53]. When acetic acid is administered, it causes the release of pro-inflammatory mediators such as cytokines, leukotrienes, prostaglandins, substance P, and bradykinin, which leads the animal to show motor nociceptive behavior based on the activation of visceral and somatic nociceptors in the peritoneum [14]. This way, compounds that can inhibit these mediators affect this model [54]. In the meantime, oral treatment with FB at all doses significantly (*p* < 0.001) suppressed the number of contortions induced by acetic acid, in a dose-dependent manner, compared to the control group.

The formalin assay was used to evaluate the antinociceptive response of FB in the neurogenic and inflammatory phases. This test is a two-phase model for assessing nociception. In the first phase, there is direct stimulation of peripheral nociceptors, such as TRPA1, with the release of substance P and glutamate; its inhibition is indicative of central analgesic drugs such as opioids and narcotics [55]. The second phase results from the release of inflammatory mediators and stimulation of nociceptors, which are inhibited by drugs that act at a peripheral level, such as COX inhibitors. Therefore, FB at all doses showed a significant effect (*p* < 0.001) in reducing paw licking time in both stages in a dose-dependent manner, but with an increased action in the second stage. Morphine was active in both phases, while indomethacin better suppressed the inflammatory response.

In the same way, research with the protein fraction of the latex and the hydroethanolic extract of the leaves of *C. procera* showed antinociceptive activity. At doses of 12.5, 25, and 50 mg·kg^−1,^ proteins extracted from the latex reduced the number of contortions by 67.9, 85 and 99.5%, respectively. In the formalin test, the doses of 12.5, 25 and 50 mg·kg^−1^ were able to have an analgesic effect in the 1st and 2nd phases, with inhibition percentages of 9.8, 42 and 66.6, 99.3%. An extract of the flowers at doses of 30, 300 and 100 mg·kg^−1^ significantly reduced (*p* < 0.001) the number of contortions caused by acetic acid, as well as the time taken to lick the paw of the mice in both experimental phases [8,56]. Therefore, this corroborates the research data from the FB and shows the antinociceptive potential of the species, with significant inhibition of the inflammatory pain stimulus.

Likewise, another study showed that the methanolic extract of *C. procera* leaves at a dose of 200 mg·kg^−1^ had a potent analgesic effect, inhibiting acetic acid-induced writhing in mice by 74.48% [57]. In addition, the ethanolic extract of the leaves showed the potential to reduce nociception in mice with a count of 16.60 ± 8.81, 40.40 ± 4.09 and 23.30 ± 9.88 twitches, compared to the control which showed 48.0 ± 4.60, at doses of 100, 200 and 400 mg·kg^−1^, respectively. For the formalin test carried out on Wistar rats, the extract at the same concentrations showed strong antinociceptive [58].

Moreover, the species *Calotropis gigantea* (Apocynaceae) has analgesic and anti-inflammatory properties, since its chemical composition contains a high amount of flavonoids such as quercetin and phenolic acids, as can be seen in this study [59]. In another study, *C. gigantea* root extract showed high antinociceptive and anti-inflammatory action (*p* < 0.001) through abdominal contortions, formalin and carrageenan-induced paw edema tests. This is due to the phytoconstituents present in this plant matrix, such as glycosides, flavonoid triterpenes and phenolic acids, which act by inhibiting cyclooxygenase, eliminating free radicals and negatively regulating Nuclear Kappa Factor B (NF-kB) [60].

Another species belonging to the Apocynaceae family and found in Brazil is *Allamanda blanchetii*, which, like the other plants mentioned, has antinociceptive action mediated by flavonoids, phenolic acids and glycosides. The extract of its leaves was tested against the acetic acid writhing model and showed a strong pain reduction potential of 55.32, 38.67 and 22.85% at doses of 100, 200 and 400 mg·kg^−1^ [61].

Moreover, the inhibitory effect on acetic acid-induced nociceptive stimuli may be attributed to the presence of flavonoids and phenolic acids in the FB. For instance, quercetin can modulate neuronal excitability in the nervous system, including nociceptive sensory transmission, by inhibiting the peripheral COX-2 signaling cascade and voltage-gated ion channels, as well as by reducing the release of pro-inflammatory cytokines (IL-1, TNF-α), which play a significant role in the inflammatory process and the development of pain [62,63].

When analyzing the effect of quercetin and isorhamnetin on inflammatory gene expression in murine macrophages stimulated with lipopolysaccharides (LPS), it was shown that they are effective in reducing TNF-α, IL-1β and IL-6 [64]. Additionally, a study revealed that caffeic acid has significant antinociceptive effects through the opioid and vanilloid pathways, with partial inhibition of the TRPV-1 receptor, and in the formalin test it reduced pain in both experimental phases [65]. Therefore, these bioactive compounds may possibly influence the results of antinociceptive activity in the peripheral and central regions.

Kaempferol, another compound present in FB, significantly reduced acute pain in acetic acid-induced writhing, formalin, and tail-flick tests used to evaluate antinociceptive activity, demonstrating a central mechanism of action through the inhibition of TRPV-1 [66]. Furthermore, through in silico analyses, it was observed that quercetin and kaempferol reduce the expression of TNF-α and Epidermal Growth Factor Receptor (EGFR), both of which act through positive feedback to enhance arachidonic acid expression, a key molecule in the inflammatory process [67]. Thus, these two flavonoids contribute to the reduction in both central pain and pain triggered by the inflammatory cascade

Additionally, roseoside, a compound isolated from the FB of *C. procera*, shows significant anti-inflammatory activity. The ability of the test sample to act in both phases of the experiment, with phase 2 being more representative, can also be attributed to the aforementioned flavonoids and phenolic acids, as well as to this substance, which has the effect of fighting inflammation mediated by inhibiting the production of NO by macrophages and the release of leukotrienes by mast cells, as well as a high effect against the production of IL-6 and moderate action against TNF-α [35,68,69]. This anti-inflammatory molecule may be associated with the antinociceptive effect observed in the acetic acid-induced writhing test, since there is an intrinsic relationship between pain and inflammation.

Therefore, based on the phytochemical profile and pharmacological data of FB, which demonstrated an absence of toxicity in the experimental assays employed and a high antinociceptive potential, it has proven to be promising for pharmacological applications. It was observed that, even at doses lower than those described in the literature, FB significantly inhibited abdominal writhing and paw licking time in mice. These findings indicate that FB is a relevant target for the development of new formulations and may represent a therapeutic alternative in pain modulation.

## 4. Materials and Methods

### 4.1. Herbal Material

**Figure 5:**
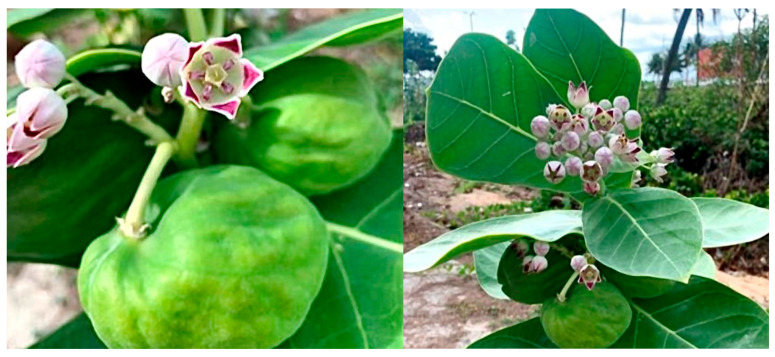
Species *C. procera*.

The species *C. procera* (Apocynaceae) was collected in January 2021, on the coast of the municipality of Cabedelo, Paraíba-Brazil (7°03′13.1″ S 34°50′35.2″ W) (Figure 5). Botanical identification was carried out with the help of researcher César Alves Carneiro from the Lauro Pires Xavier Herbarium at the Federal University of Paraíba, where an exsiccate was deposited under catalog number JPB0066233. The selected species and the proposed project are registered in the National System for the Management of Genetic Heritage and Associated Traditional Knowledge (SisGen) under the code A9852FB.

### 4.2. Obtaining the Crude Ethanolic Extract (CEE)

The botanical material was dehydrated in an air circulation oven at 45 °C for 72 h. It was then processed in a mechanical mill to obtain 629.25 g of the dried and crushed plant drug, which was subjected to maceration with 95% ethanol (EtOH) for 72 h. The resulting extractive solution was concentrated under reduced pressure in a rotary evaporator (IKA RV 3 Eco, Staufen, Germany) at 55 °C to obtain the crude ethanolic extract (CEE).

### 4.3. CEE Fractionation by Partitioning

The CEE was defatted with hexane, in which this solvent was added to the extract, then the hexane solution was taken to a mechanical shaker and finally filtered. Subsequently, 55 g of the defatted extract was solubilized in an ethanol/water solution (7:3 *v*/*v*) in order to obtain a hydroalcoholic solution. This was subjected to liquid–liquid partitioning using solvents in an increasing polarity gradient three times. The extractive solutions of the fractions were concentrated in a rotary evaporator under reduced pressure at a temperature of 50 °C to obtain the fractions: hexanic, chloroformic, ethyl acetate and *n*-butanolic (FB) (4.345 g), the latter being the object of the research, since there are no reports of pharmacological studies on it, as well as few chemical references, being a fraction not yet studied by our group [70]. The yield of the fraction FB was determined using the equation: Fraction yield (%) = (mass of the fraction (g)/mass of the crude extract (g)) × 100, which resulted in a yield of 7.9%.

### 4.4. Solid Phase Extraction (SPE)

A solid phase extraction of 1.5 g of the FB was carried out, which was suspended in water/methanol, centrifuged for 20 min at 5000 rpm (ROTINA-380 R, Tuttlingen, Germany) and filtered (PTFE syringe filter 13 mm × 0.22 μm). A polypropylene cartridge containing the sorbent, silica C-18 (Strata-C18-E, Torrance, CA, USA), conditioned with methanol and distilled water (1:9) was used for the chromatographic process. The sample was placed on top of the cartridge and for elution a binary mixture of organic solvents, aspirated with the aid of a vacuum pump (TECNAL–TE-0581, São Paulo, SP, Brazil) operating at 600 mmHg, was used, starting with 95% distilled water to 100% MeOH. The 10 fractions obtained were concentrated in a rotary evaporator.

### 4.5. Phytochemical Characterization by HPLC-ESI-MS/MS

For ESI-MS^n^ analysis in negative mode, a sample solution was initially prepared at a concentration of 1 mg·mL^−1^ and injected into a UFLC (ShimadzuC, Kyoto, Japan, JP) with two LC20AD pumps, a SIL20AHT autosampler and a CBM20A system controller, coupled to an Ion-Trap mass spectrometer (AmaZon X, Bonn, Germany). The Ion-Trap parameters were: capillary voltage set at 4.5 kV, temperature set at 200 °C; drying gas flow rate (N_2_) of 8 mL·min^−1^ and nebulizer pressure of 58.02 psi. CID fragmentation was carried out in auto MS/MS mode using the advanced resolution mode for MS and MS/MS mode and the spectra (*m*/*z* 50–1500) were recorded every 2 s.

The liquid chromatography system used a YMC-Triart C-18 column (250 mm × 4.6 mm and 5 μm particles) and ultrapure acidified water (0.1% formic acid) and chromatographic grade methanol as mobile phases A and B, respectively. The following elution method was used, at a flow rate of 0.6 mL·min^−1^, in which 0.01–60 min—5–100% of B, 60–80 min—100–100% of B, 80–85 min—100–5% of B. The relevant chromatogram is found in the Supplementary Material and the compounds were tentatively annotated by comparing the corresponding fragmentation patterns (MS^2^ and MS^3^) with data reported in the scientific literature.

### 4.6. Isolation and Characterization of Compounds

FB3 (102.9 mg), obtained through solid-phase extraction, was selected for preparative HPLC, with the following parameters: solvent A = acidified water (0.1% formic acid); solvent B = chromatographic grade methanol, at a flow rate of 0.6 mL·min^−1^, using the following gradient: 0.01–5 min—5–40% of B, 5–30.0 min—40–43% of B, 30.0–35.0 min—43–100% of B, 35.0–55.0 min—100–100% of B, 55.0–60.0 min—100–5% of B, 60.0–80.0 min—5–5% of B. Ten injections were performed using a 100 µL loop, and four fractions were collected.

Fraction 4, designated as CP-R, was characterized by ^1^H and ^13^C Nuclear Magnetic Resonance (NMR) spectra. The chromatogram from the HPLC analysis and further details on the characterization of the fraction can be found in the Supplementary Material.

### 4.7. Preparing the Substances

For the pharmacological tests, the FB was dissolved in a saline solution containing Tween^®^ 80 (0.1%) before use and administered according to the route required for the procedure. Additionally, as a negative control, the vehicle used to dissolve the sample (saline solution (0.9%) with Tween^®^ 80 (0.1%)) was assigned, while morphine and indomethacin were used as positive controls. For the administration of the substances, the standard dose was 100 µL/10 g b.w.

### 4.8. Ethical Procedures and Vivarium Conditions

The experimental protocols were approved by the Ethics Committee on the Use of Animals (CEUA) of the University Center—UNIFACISA under approval number 03.0001.2024/05.2024, with the standards approved by the National Council for the Control of Animal Experimentation (CONCEA). For the pharmacological tests, adult male and female (only for the acute oral toxicity test) Swiss albino mice (*Mus musculus*) weighing between 28 and 31 g and aged 50–54 days were used. They were housed under conventional circumstances, which included a 12 h light/dark cycle, a temperature range of 22–2 °C and a humidity of 50–55%. They also received *ad libitum* access to filtered water and standard rodent chow. The animals were euthanized by hyperdosing the combination of ketamine 10% (300 mg·kg^−1^) and xylazine 2% (30 mg·kg^−1^) intraperitoneally as recommended by the 2018 CONCEA Euthanasia Practice Guidelines.

### 4.9. Hemolytic Activity Test

The experimental procedure consisted of collecting blood from mice in tubes containing ethylene diaminetetraacetic acid (EDTA). After centrifugation, the plasma was separated and the red blood cells (RBC) were washed three times with saline solution to obtain a 5% RBC suspension. This was then added to test tubes containing 1.0 mL of FB solutions at concentrations of 2000, 1000, 500, 250 and 125 µg·mL^−1^. After 1 h, the tubes were centrifuged and the supernatant was analyzed in a Shimadzu UV-1900 spectrophotometer (Kyoto, Japan, JP) at 540 nm. Triton X-100 (Sigma Aldrich, St. Louis, MO, USA) at 1% was used as a 100% hemolysis control and saline served as a blank (0% hemolysis). The analysis was carried out in triplicate, and the hemolytic potential (HP) of the substances was calculated using the equation: HP = (Ae − Ab)/At × 100, where HP represents the Hemolytic Potential (in percentage), Ae is the absorbance of the red blood cells treated with the extract, Ab is the absorbance of the blank, and At is the absorbance of the red blood cells treated with Triton X-100 [17,45].

### 4.10. Acute Oral Toxicity Assay

The acute oral toxicity test on mice was carried out in accordance with the Organization for Economic Cooperation and Development (OECD), n°. 423. For the toxicity test, two groups (n = 3) of female Swiss mice were orally administered saline solution (0.9%) with Tween^®^ 80 (0.1%) or 300 mg·kg^−1^ of FB. Possible behavioral alterations suggestive of activity on the Central Nervous System (CNS) or Autonomic Nervous System (ANS) were evaluated after the administration of FB, and careful observation of the effects of the substance was carried out to detect general toxic signs in the first 4 h after administration and once a day, always at the same time until the 14th day. In addition, parameters such as water consumption, food intake and weight evolution were observed throughout the 14 days of the test. On the 15th day, the animals were weighed and anesthetized (xylazine 5 mg·kg^−1^ and ketamine 100 mg·kg^−1^) to collect blood by cardiac puncture for hematological and biochemical analysis using specific kits and then euthanized.

#### 4.10.1. Evaluation of Hematological Parameters

Hematological analyses were carried out immediately after blood collection using an automatic hematological analyzer (Coulter STKS, Beckman Coulter, Miami, FL, USA) and optical microscopy. The red blood cell (RBC) and white blood cell (WBC) counts, hemoglobin (HB), hematocrit (HCT), mean corpuscular volume (MCV), mean corpuscular hemoglobin (MCH), mean corpuscular hemoglobin concentration (MCHC), red blood cell distribution range (RDW) and differential leukocyte count (segmented (SEG), lymphocytes (LIN), monocytes (MON), basophils (BASO) and eosinophils (EOS)) were added.

#### 4.10.2. Evaluation of Biochemical Parameters

The blood was centrifuged for the biochemical assessment of albumin (ALB), alanine aminotransferase (ALT), Alkaline phosphatase (ALP), aspartate aminotransferase (AST), bilirubin (BIL), gamma-glutamyl transferase (GGT), total protein (TP), urea (UR) and creatinine (CRE). These markers were measured using specific kits (Labtest Diagnostic, Lagoa Santa, Brazil) and a COBAS Mira Plus analyzer (Roche Diagnostics Systems, Basel, Switzerland) [71].

### 4.11. Assessment of Antinociceptive Activity

#### 4.11.1. Acetic Acid Induced Writhing Test

Male mice were divided into 5 groups (n = 6) and treated orally with saline solution (0.9%) with Tween^®^ 80 (0.1%), FB (3.75, 7.5 or 15 mg·kg^−1^ o.r) or indomethacin (20 mg·kg^−1^, i.p). Each animal received an intraperitoneal injection (0.1 mL/10 g) of 0.85% (*v*/*v*) acetic acid in saline solution and was placed in a polyethylene box to record the number of complete contortions (elongation of the front and hind legs) in the interval corresponding to 5–15 min after the injection of acetic acid [71].

#### 4.11.2. Formalin Test

Groups of 6 male mice (n = 6) received saline solution (0.9%) with Tween^®^ 80 (0.1%) (0.9%), FB (3.75, 7.5 or 15 mg·kg^−1^ o.r), morphine (10 mg·kg^−1^ i.p.) or indomethacin (20 mg·kg^−1^ i.p.) orally. After 60 min, 20 µL of 2.5% (*v*/*v*) formalin in saline were injected into the subplantar region of the right hind paw of each animal. The time spent by the mouse licking the paw was recorded during the first 5 min after the formalin injection (first phase: neurogenic pain), as well as 15 to 30 min after the injection (second phase: inflammatory pain) [72,73].

### 4.12. Data Analysis

The results were expressed as mean ± standard media error (SEM). To analyze the data obtained in the acute toxicity tests, the unpaired Student’s *t*-test was used to compare 2 groups. For the other experimental protocols, one-way analysis of variance (ANOVA) was used, followed by Tukey’s post-tests for multiple comparisons. *p* values of less than 0.05 (*p* < 0.05) were considered significant.

## 5. Conclusions

When investigating the phytochemical profile of FB from the leaves of *C. procera*, it was possible to verify the presence of phenolic acids, flavonoids, and the unprecedented isolation of roseoside in the species. When administered orally in a single dose, it proved to be safe up to a dose of 300 mg·kg^−1^, as well as revealing an antinociceptive effect, mainly related to inflammatory pain, with the possible involvement of the metabolites found in its composition, which justifies its extensive ethnopharmacological use. This study provides data for future research into the isolation of bioactive compounds and enables new research to analyze their intrinsic inhibitory effect on nociception, as well as possible mechanisms of action that justify their activity.

## Figures and Tables

**Table 1:** Phytochemical profile of FB from *C. procera* leaves by HPLC-ESI-MS/MS.

Peak	R. T. (min)	UV λmax (nm)	Molecular Weight	[M–H]^−^ *m*/*z*	MS^2^/MS^3^	Annotation	Ref.
1	4.9	258	378	377 (341-H+Cl)	MS^2^ [377]: 341 (100)/MS^3^ [377 → 341]: 281 (11.35); 251 (16.78); 179 (100); 161 (25.79)	Caffeic acid-*O*-hexoside (Chlorine Adduct)	[20]
2	7.8	-	268	267	MS^2^ [267]: 223 (100); 211 (94.92)/MS^3^ [267 → 223]: 281 (11.35); 251 (16.78); 179 (100); 161 (25.79)	Methyldaidzein	[21]
3	8.2	237.324	432	431 (385-H+Formate)	MS^2^ [431]: 385 (100)/MS^3^ [431 → 385]: 223 (96.48); 205 (100); 179 (7.08); 153 (73.71)	Roseoside (Formate Adduct)	[22,23,24]
4	12.8	256.350	610	609	MS^2^ [609]: 343 (8.64); 301 (100); 271 (11.12); 255 (6.81)/MS^3^ [609 → 301]: 271 (100); 255 (59.75); 243 (10.72); 179 (91.80); 151 (71.09)	Quercetin-*O*-rutinoside	[25]
5	13.0	-	180	179	MS^2^ [179]: 135 (100)/MS^3^ [179 → 135]: 91 (100)	Caffeic acid	[26]
6	14.7	255.353	594	593	MS^2^ [593]: 327 (5.23); 285 (100); 257 (6.03); 255 (5.59)/MS^3^ [593 → 285]: 267 (68.95); 257 (100); 255 (34.03); 241 (34.11); 229 (43.36); 213 (34.12); 197 (29.46); 163 (19.16); 151 (5.33)	Kaempferol-*O*-rutinoside	[27]
7	15.0	254.355	624	623	MS^2^ [623]: 315 (100); 300 (40.18); 271 (21.70); 255 (12.45); 243 (3.32)/MS^3^ [623 → 315]: 301 (20.58); 299 (100); 287 (5.74); 272 (13.35); 255 (5.60)	Isorhamnetin-*O*-rutinoside	[28]

**Table 2:** Consumption of the animals submitted to the toxicity test, treated with the by the FB (300 mg·kg^−1^), for 14 days.

Parameters	Treatment	
	Control	FB (300 mg·kg^−1^)
Food consumed (g)	14.93 ± 0.77	17.43 ± 0.56
Water consumed (mL)	24.00 ± 1.00	26.57 ± 1.07
Mean weight (g)	27.98 ± 0.37	30.50 ± 0.27

The values represent the mean ± SEM (n = 3/group). No significant differences were found (*p* > 0.05) compared to the control group.

**Table 3:** Hematological parameters of mice treated with FB o.r (300 mg·kg^−1^).

Parameters	Treatment	
	Control	FB (300 mg·kg^−1^)
RBC	5.78 ± 0.36	5.95 ± 0.48
HCT	34.19 ± 3.66	35.28 ± 3.29
HBG	13.97 ± 0.42	13.67 ± 0.68
MCV	42.15 ± 4.11	45.07 ± 3.73
MCH	15.03 ± 0.72	15.21 ± 0.84
MCHC	34.16 ± 3.85	32.16 ± 3.24
WBC	7.55 ± 0.92	7.15 ± 0.66
SEG	60.09 ± 5.75	57.17 ± 5.54
LIN	37.10 ± 3.02	39.84 ± 3.24
MON	2.19 ± 0.24	2.21 ± 0.26
BASO	0.16 ± 0.04	0.19 ± 0.04
EOS	0.54 ± 0.07	0.59 ± 0.04

RBC: Red blood cells (10^6^/mm^3^); HCT: Hematocrit (%); HBG: Hemoglobin (g/dL); MCV: Mean Corpuscular Volume (%); MCH: Mean Corpuscular Hemoglobin (%); MCHC: Mean Corpuscular Hemoglobin Concentration (%); WBC: White Blood Cells (10^3^/mm^3^); SEG: Segmented (%); LIN: Lymphocytes (%); MON: Monocytes (%); BASO: Basophils; EOS: Eosinophils. The values represent the mean ± SEM (n = 3/group). No significant differences were found (*p* > 0.05) compared to the control group.

**Table 4:** Biochemical parameters of mice treated with FB o.r (300 mg·kg^−1^).

Parameters	Treatment	
	Control	FB (300 mg·kg^−1^)
ALB	3.09 ± 0.41	3.70 ± 0.37
ALP	67.01 ± 5,55	70.24 ± 6.11
AST	74.05 ± 6.13	70.21 ± 7.08
ALT	44.87 ± 4.01	47.05 ± 4.32
BIL	0.62 ± 0.09	0.66 ± 0.05
GGT	19.04 ± 1.22	21.83 ± 1.87
PT	7.32 ± 0.73	6.91 ± 0.84
UR	35.64 ± 2.14	33.77 ± 2.43
CRE	0.93 ± 0.11	0.88 ± 0.14

ALB: Albumin (g/dL); ALT: Alanine aminotransferase (U/L); AST: Aspartate aminotransferase (U/L); ALP: Alkaline phosphatase (U/L); BIL: Bilirubin (mg/dL); GGT: Gamma Glutamyl Tranferase; PT: Total protein (g/dL); UR: Blood urea (mg/dL); CRE: Creatinine (mg/dL). Values represent the mean ± SEM (n = 3/group). No significant differences were found (*p* > 0.05) compared to the control group.

## Data Availability

Data are contained within the article and Supplementary Materials.

## Supplementary Materials

The following supporting information can be downloaded at https://www.mdpi.com/article/10.3390/plants14233622/s1, Figure S1. Chromatogram of FB from *C. procera* leaves at a wavelength of 254 nm; Figure S2. Chromatogram of FB3 in preparative HPLC, Nuclear Magnetic Resonance Spectra ^1^H e ^13^C, Structural determination of CP-R; Table S1. NMR data (*J* in Hz and δ in ppm, 400 MHz for ^1^H and 100 MHz for ^13^C) of CP-R compared with literature [42] Table S2. Two-dimensional NMR data (*J* in Hz and δ in ppm, 400 MHz for ^1^H and 100 MHz for ^13^C) of CP-R compared with literature [38,39]; Figure S3. ^1^H NMR spectrum (400 MHz, DMSO-d_6_) of CP-R; Figure S4. Expansion of the ^1^H NMR spectrum (400 MHz, DMSO-d_6_) of CP-R in the region of 0.6–2.7 ppm; Figure S5. Expansion of the ^1^H NMR spectrum (400 MHz, DMSO-d_6_) of CP-R in the region of 2.7–4.8 ppm; Figure S6. Expansion of the ^1^H NMR spectrum (400 MHz, DMSO-d_6_) of CP-R in the region of 4.8–7.0 ppm; Figure S7. ^13^C-BB NMR spectrum (100 MHz, DMSO-d_6_) of CP-R; Figure S8. ^1^H-^13^C–HSQC contour map (400 × 100 MHz, DMSO-d_6_) of CP-R; Figure S9. ^1^H-^13^C-HMBC contour map (400 × 100 MHz, DMSO-d_6_) of CP-R.
