# Supplementary material for: Phytochemical Profile, Toxicity Evaluation and Antinociceptive Effect of the n-Butanolic Fraction from the Leaves of Calotropis procera (Aiton) W.T Aiton (Apocynaceae)
Source: Plants (Basel). 2025 Nov 27;14(23):3622. doi: 10.3390/plants14233622 (PMC12694354; doi:10.3390/plants14233622)
Supplement: Supplementary file 1 [file plants-14-03622-s001.zip › plants-3983363-supplementary.pdf]

Supplementary Material

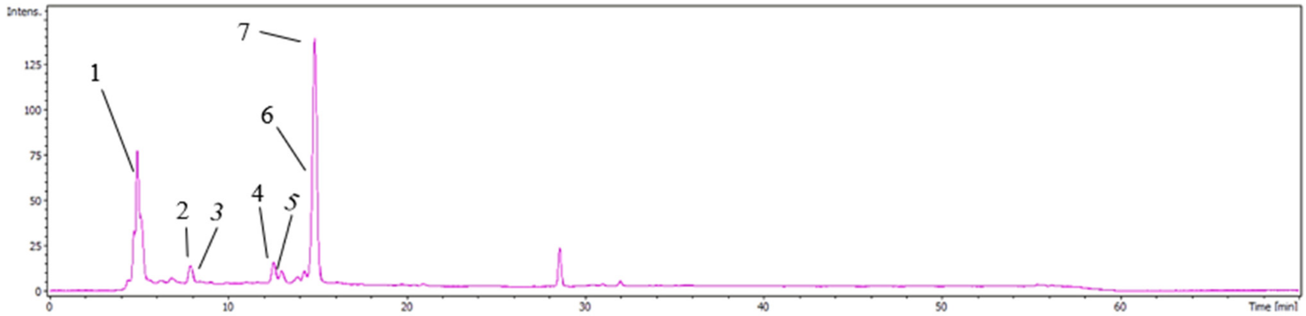

Figure S1. Chromatogram of FB from *C. procera* leaves at a wavelength of 254 nm.

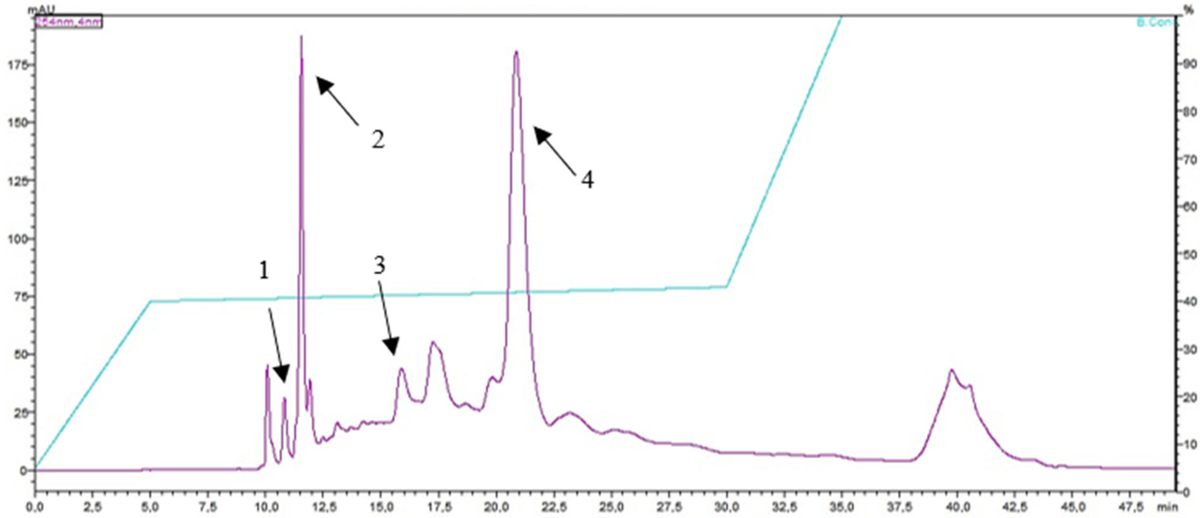

Figure S2. Chromatogram of FB3 in preparative HPLC.

Nuclear Magnetic Resonance Spectra ^1H e ^{13}C

The NMR spectra of **fraction 4**, obtained from preparative scale chromatography and coded as CP-R, were recorded on Brucker equipment operating at 400 MHz (^1H) and 100 MHz (^{13}C). Deuterated solvents (dimethyl sulfoxide - DMSO-d_6) will be used to dissolve the samples. The chemical shifts (δ) will be expressed in parts per million (ppm) and the coupling constants J in Hz, using the conventions *s* (singlet), *d* (duplet), *dd* (double duplet), *ddd* (double duplet of double duplet), *t* (triplet) and *m* (multiplet) to indicate the multiplicities of the ^1H signals.

Table S1. NMR data (*J* in Hz and δ in ppm, 400 MHz for ^1H and 100 MHz for ^{13}C) of CP-R compared with literature (Tpsomo, 2010) .

Posição	1. **CP-R**		2. **Tsopmo, 2010***	
	**δ_H**	**δ_C**	**δ_H**	**δ_C**
**1**	-	40.96	-	42.4
**2a**	2.05 (1H, *d*, *J* = 16.8)		2.50 (1H, *d*, *J* = 17.0)	
		49.39		50.7
**2b**	2.41 (1H, *d*, *J* = 16.8)		2.12 (1H, *d*, *J* = 17.0)	
**3**	-	197.5	-	201.2
**4**	5.76 (1H, *s*)	125.7	5.85 (1H, *s*)	127.1
**5**	-	164.1	-	167.3

**6**	-	77.9	-	80.7
**7**	5.74 (1H, *d*, *J* = 5.2)**	130.3	5.83 (1H, *d, J* = 4.2)**	131.5
**8**	5.78 (1H, *t*)	133.3	5,82 (1H, *d*, *J* = 4.2)	135.2
**9**	4.32 (1H, *q*, *J* = 6.4)	73.7	4.39 (1H, *m*)	76.8
**10**	1.18 (3H, *d*, *J* = 6.4)	20.9	1.27 (3H, *d*, *J* = 7.0)	21.1
**11**	0.93 (3H, *s*)	23.1	1,.3 (3H, *s*)	23.4
**12**	0.92 (3H, *s*)	24.1	1.03 (3H, *s*)	24.7
**13**	1.81 (3H, *s*)	18.9	1.91 (3H, *s*)	19.5
**1'**	4.16 (1H, *d*, *J* = 7.8)	100,9	4.37 (*t*, *J* = 8.7)	101,2
**2'**	2.90 – 4.0 (*m*)	74,6	3.91 (1H, *d*, 1.2)	79,2
**3'**	2.90 – 4.0 (*m*)	76,8	-	78,6
**4'**	2.90 – 4.0 (*m*)	70,0	4,01 (1H, *d*, *J* = 10.5)	71,7
**5'**	2.90 – 4.0 (*m*)	76,8	3,61 (2H, *d*, *J* = 2.2)	77,9
**6'**	2.90 – 4.0 (*m*)	61,1	-	62,8

*500 MHz, CD^3OD. **Second coupling constant not obtained due to overlap.

Table S2. Two-dimensional NMR data (*J* in Hz and δ in ppm, 400 MHz for ^1H and 100 MHz for ^{13}C) of CP-R compared with literature (Nascimento, 2019; Wang et al., 2022).

	**HMBC**	**Nascimento, 2019**	**Wang *et al.*, 2022**
**Posição**	**δ_H x δ_C**	**δ_H x δ_C**	**δ_H x δ_C**
**2**	C-3	C-3	C-4, C-6
**7**	C-6, C-9	-	-
**8**	C-6, C-9	-	-
**9**	C-7, C-8, C-1'	-	C-7, C-1'
**10**	C-8, C-9	C-8, C-9	-
**11**	C-1, C-2, C-6	C-1, C-2, C-6	C-1, C-2, C-6
**12**	C-1, C-2, C-6	C-1, C-2, C-6	C-1, C-2, C-6
**13**	C-3,C-4, C-5, C-6	C-4, C-5, C-6	C-4, C-5, C-6
**1'**	C-9, C-3', C-4'	C-9	C-9

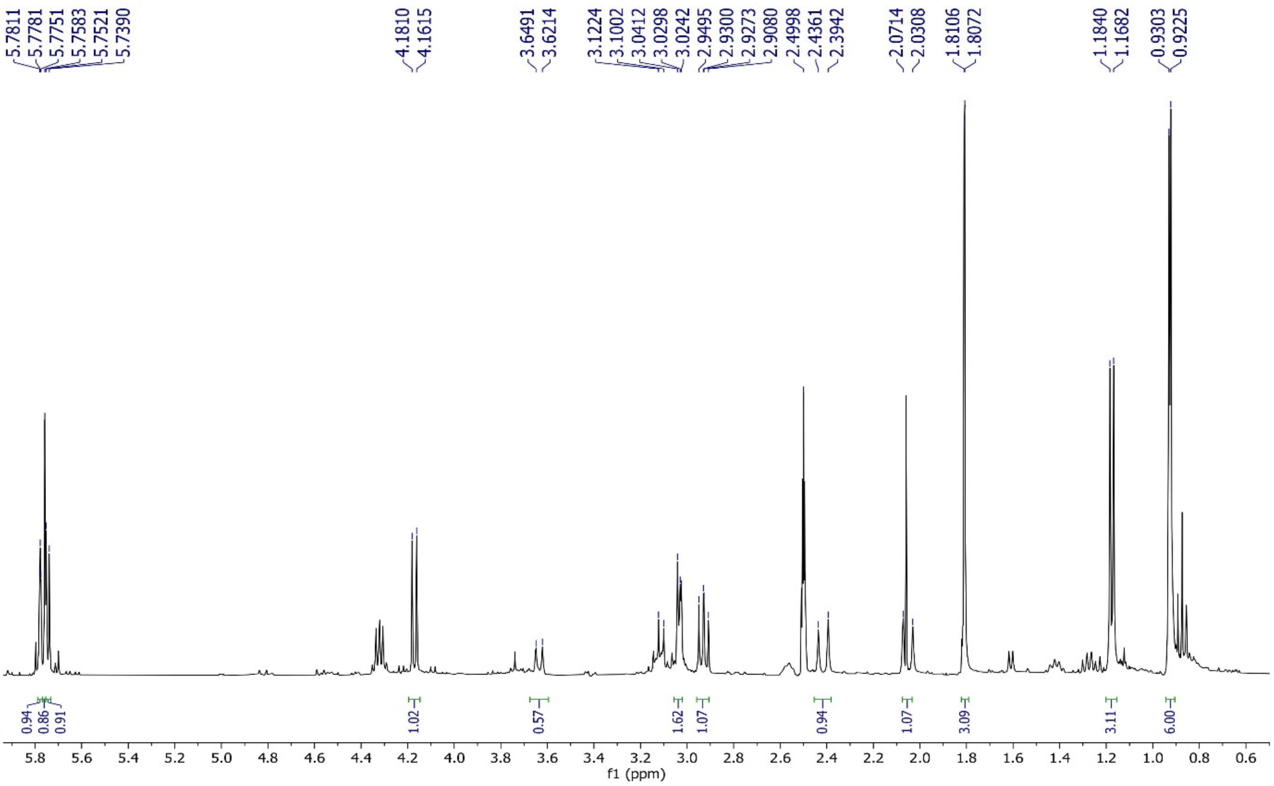

Figure S3. ^{1}H NMR spectrum (400 MHz, DMSO-d_6) of CP-R.

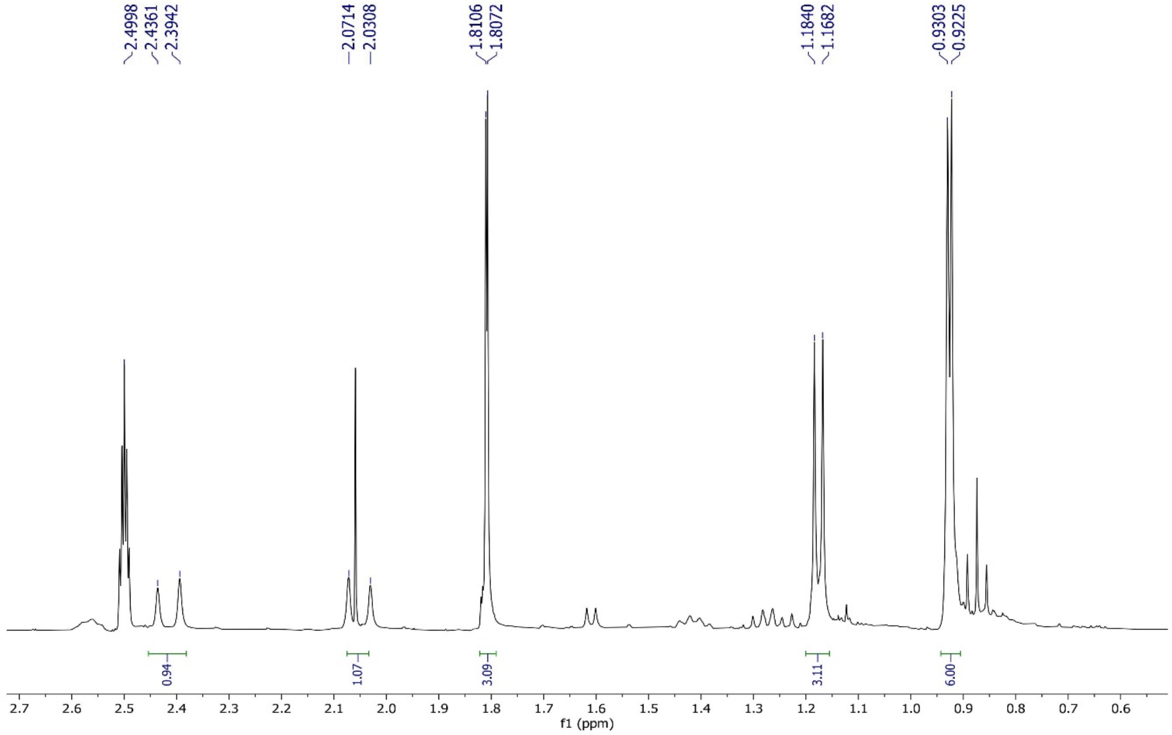

Figure S4. Expansion of the ^{1}H NMR spectrum (400 MHz, DMSO-d_6) of CP-R in the region of 0.6 - 2.7 ppm.

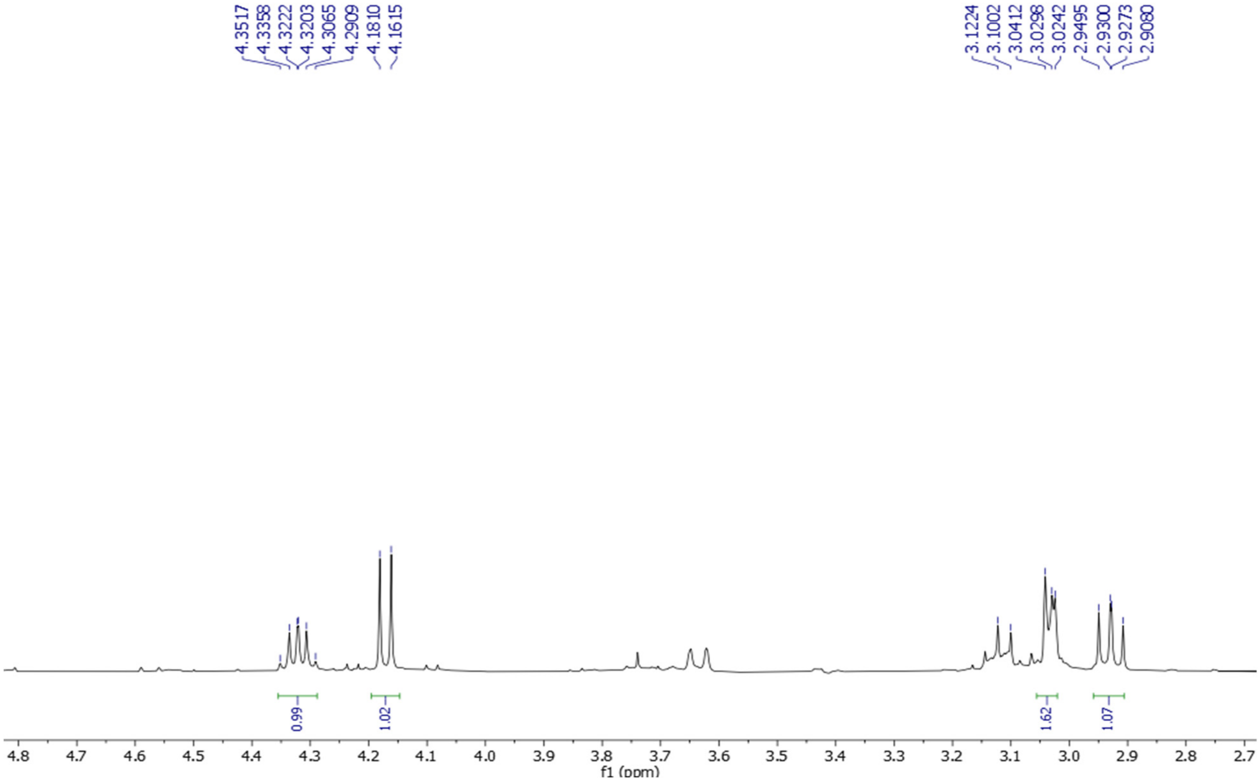

Figure S5. Expansion of the ^{1}H NMR spectrum (400 MHz, DMSO-d_6) of CP-R in the 2.7 - 4.8 ppm region.

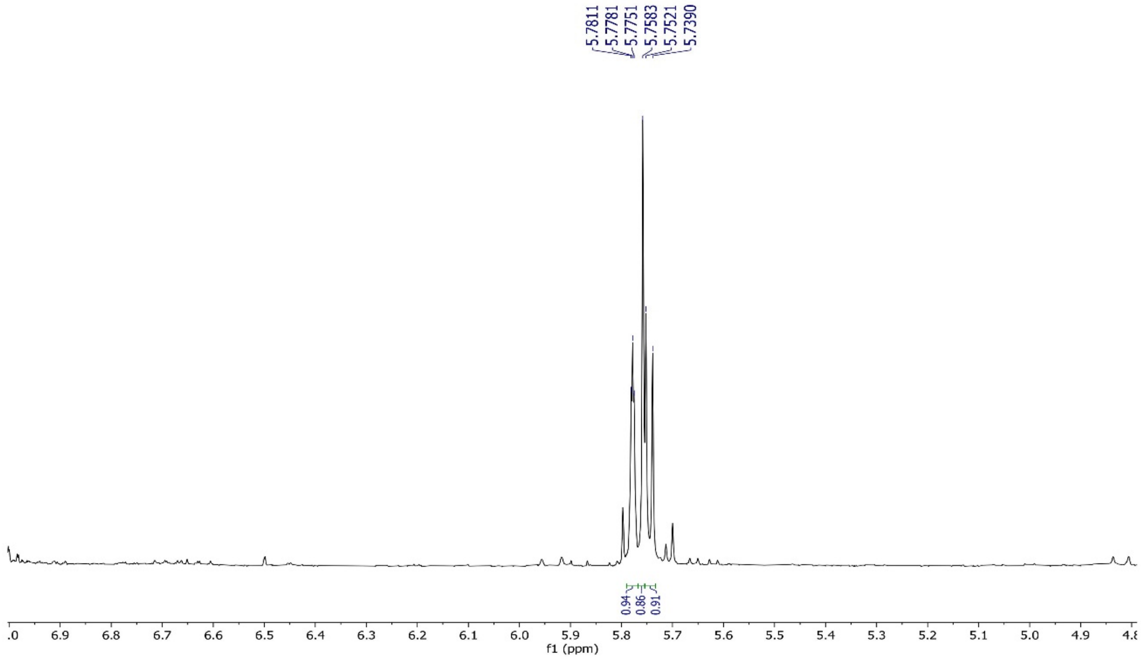

Figure S6. Expansion of the 1H NMR spectrum (400 MHz, DMSO-d_6) of CP-R in the 4.8 - 7.0 ppm region.

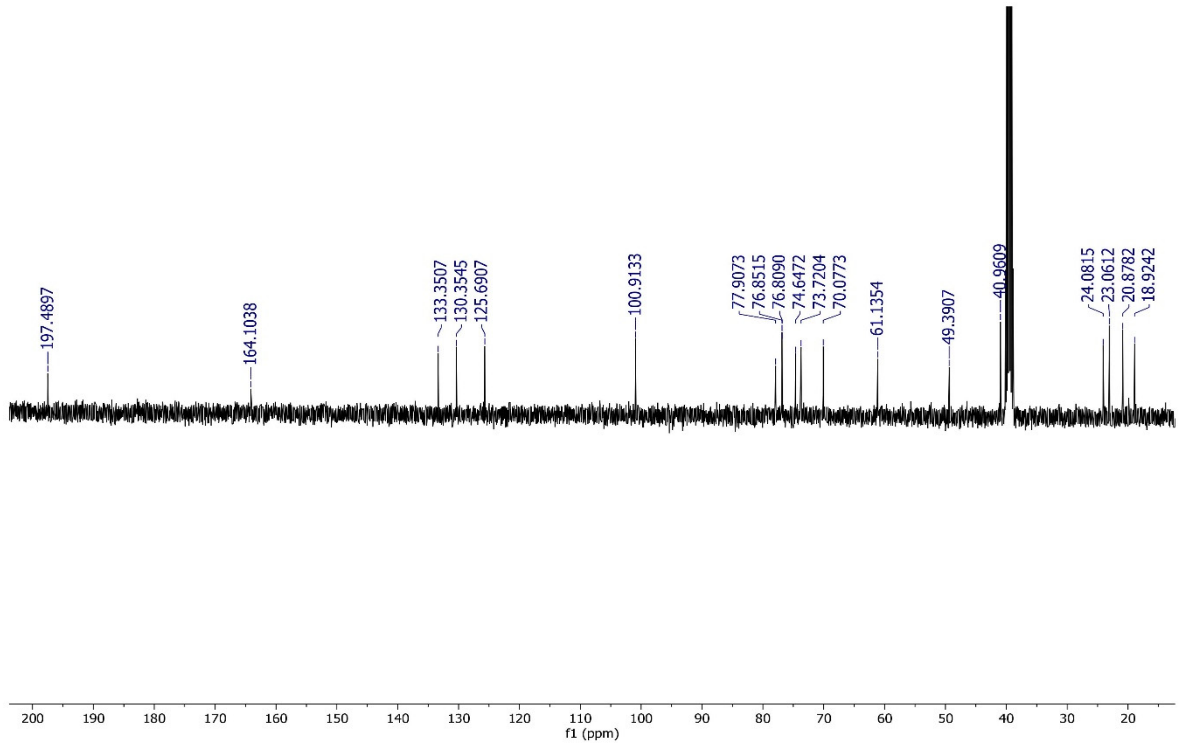

Figure S7. ^{13}C-BB NMR spectrum (100 MHz, DMSO-d_6) of CP-R.

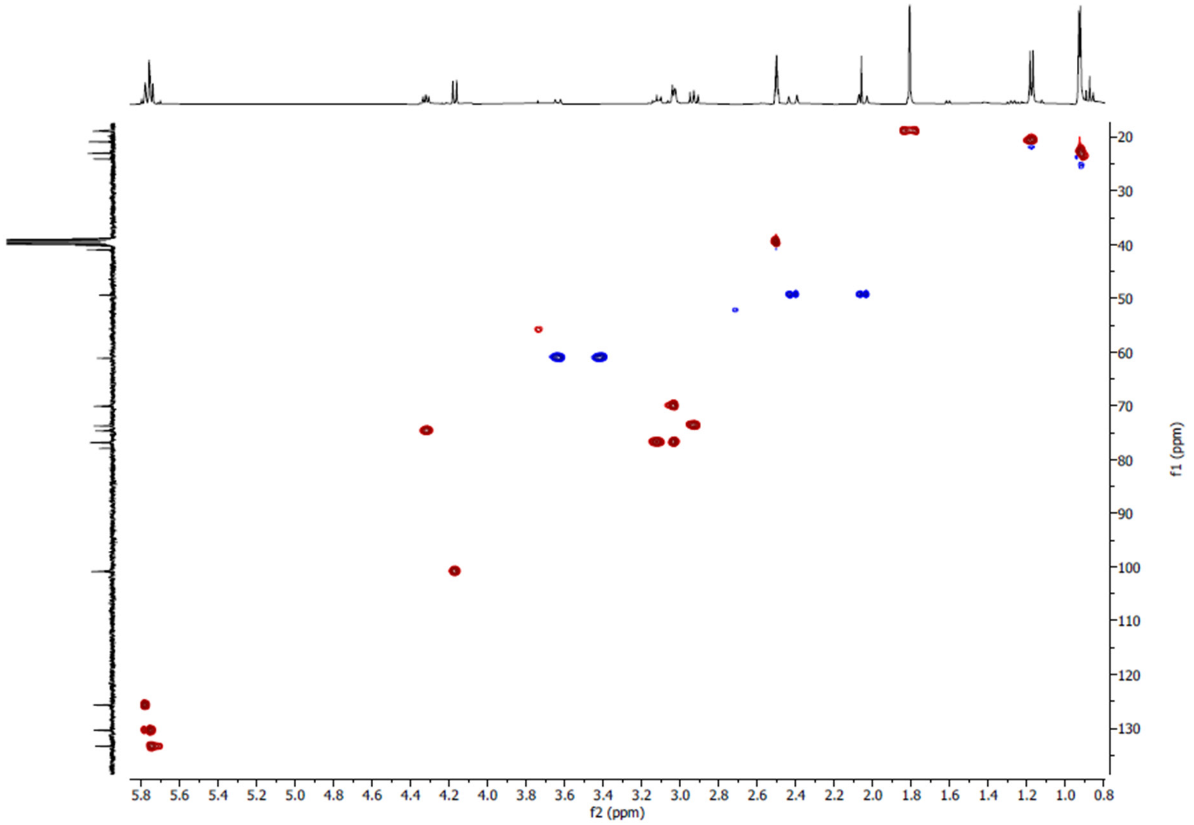

Figure S8. ^{1}H -^{13}C - HSQC contour map (400 × 100 MHz, DMSO-d_6) of CP-R.

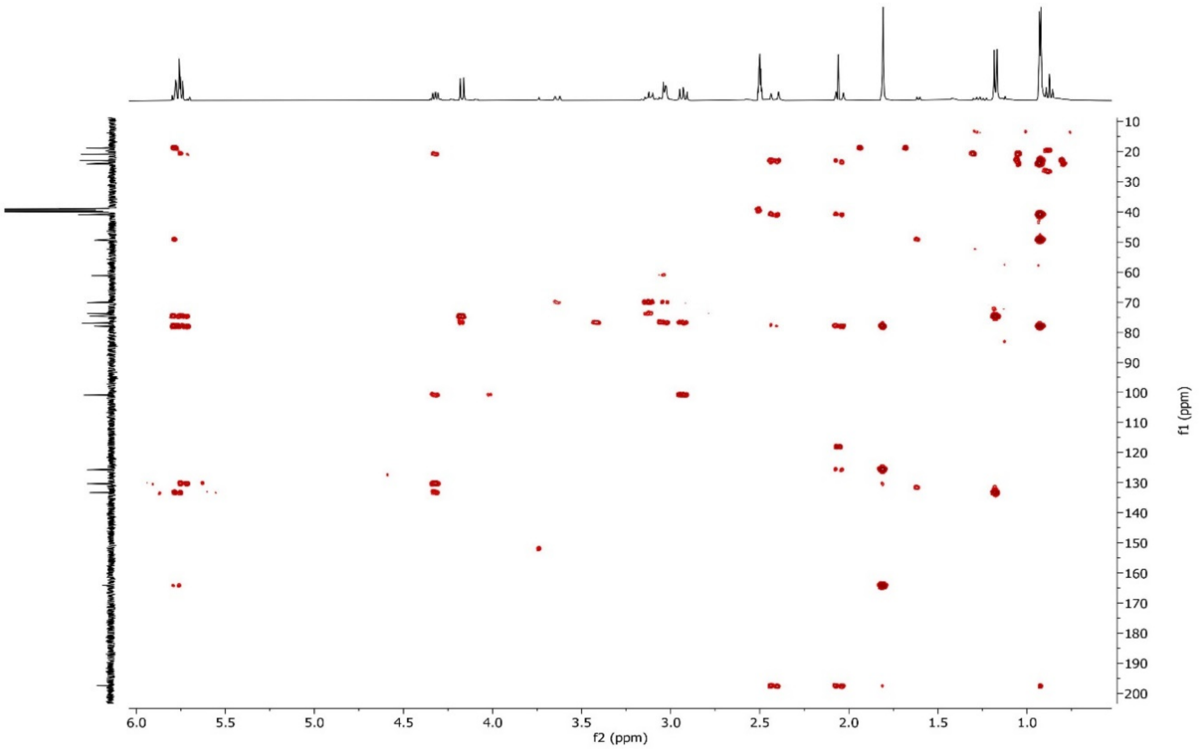

Figure S9. ^{1}H -^{13}C - HMBC contour map (400 × 100 MHz, DMSO-d_6) of CP-R.
